# Autophagy in the HTR-8/SVneo Cell Oxidative Stress Model Is Associated with the NLRP1 Inflammasome

**DOI:** 10.1155/2021/2353504

**Published:** 2021-03-27

**Authors:** Meihe Li, Tao Sun, Xiaoling Wu, Peng An, Xili Wu, Huimin Dang

**Affiliations:** ^1^Department of Traditional Chinese Medicine, Second Affiliated Hospital of Xi'an Jiaotong University, Xi'an 710004, China; ^2^Beijing Traditional Chinese Medicine Hospital Affiliated to Capital Medical University, Beijing 100010, China; ^3^Department of Pharmacy, The Second Affiliated Hospital of Air Force Medical University, Xi'an, Shaanxi 710038, China; ^4^Department of Obstetrics and Gynecology, Second Affiliated Hospital, Medical School of Xi'an Jiaotong University, China

## Abstract

We investigated whether there was activation of NLRP1 inflammasomes and excessive autophagy in oxidative stress damage. And we further demonstrate whether there is a cascade relationship between the activation of NLRP1 inflammasomes and the phenomenon of excessive autophagy. To observe the expression level of the NLRP1 inflammasome group in the pathological process of trophoblast cell oxidative stress, western blot, immunofluorescence, and qRT-PCR were performed. Autophagy in trophoblast cells after the action of H_2_O_2_ was detected by using normal trophoblast cells' NLRP1-specific activator (MDP) as a positive control. The presence of excessive autophagy was determined by comparing it with the autophagy-related proteins in normal trophoblast cells. Through siRNA-NLRP1, we investigated the role of oxidative stress and the NLRP1 inflammasome in autophagy in cells. 100 *μ*mol MDP for 24 hours can be used as the optimal concentration of the NLRP1 activator. In human placental trophoblast oxidative stress, the model group significantly increased the expression level of inflammasome IL-1*β*, CASP1, and NLRP1, compared with the control group NLRP3, and LC3-II, Beclin-1, ATG5, ATG7, and p62 overactivated the autophagy ability of cells. After the activation of NLRP1, the expression of these inflammasomes increased, accompanied by the decrease in autophagy. After the expression of NLRP1 was silenced by RNAi, the expression of inflammasome IL-1*β*, CASP1, and NLRP3 was also decreased. Still, the autophagy level was increased, which was manifested by the high expression of LC3-II, Beclin-1, ATG5, and ATG7 and the decrease in p62. Trophoblast cells showed the expression of NLRP1 protein and excessive autophagy under oxidative stress. Simultaneously, the NLRP1 inflammasome of trophoblast cells in the state of oxidative stress was correlated with autophagy. Inflammasome activation and autophagy were shown to be linked and to influence each other mutually. These may also provide new therapeutic targets in a pathological pregnancy.

## 1. Introduction

Pregnancy is considered a kind of allotransplantation process. The balance of anti-inflammatory and proinflammatory at the maternal-fetal interface plays an essential role in maintaining pregnancy. Oxidative stress refers to the state of imbalance in which reactive oxygen species (ROS) generation exceeds the antioxidant defense capacity in vivo [1]. The presence of excessive amounts of ROS can damage the lipids, proteins, or DNA of cells, thereby inhibiting their normal function [2]. Oxidative stress is related to many human diseases. There is increasing evidence that oxidative stress is closely related to the occurrence of pathological pregnancy outcomes such as abortion and preeclampsia [3]. The body's oxidation and antioxidant functions are relatively balanced during a normal pregnancy, and no oxidative stress is present [4]. However, the oxidative damage of human trophoblasts caused by any mechanism will lead to decreases in proliferation and invasiveness, abnormal placenta formation and development, and finally a pathological pregnancy [5].

Recently studies have shown that ROS produced in oxidative stress can induce autophagy [6]. Autophagy mainly occurs in eukaryotic cells, generally at a low level, and mainly maintains cell self-renewal. However, the autophagy level increases when the external environment is stimulated (e.g., by hunger, lack of nutrition, or lack of oxygen), with cells responding by recycling nutrients, which is beneficial to cell survival. Cell stimulation activates the autophagy pathway, and a small amount of autophagy lysosomes will degrade damaged proteins and organelles and resist the damage caused by external stimulation to cells. However, autophagy will lead to the self-digestion of the normal components of cells and cause cell death. Therefore, autophagy is also an intracellular regulatory mechanism that exerts opposing effects. The inflammasome is a kind of cellular multiprotein oligomer that is involved in immune and inflammatory responses. After activation, it can mediate CASP1 to transform the precursors IL-1*β* and IL-18 into mature inflammatory factors. There is increasing concern about the relationship between autophagy and apoptosis. Inflammatory corpuscles can specifically recognize intracellular pathogen-related and endogenous damage-related molecular patterns, activate CASP1, and promote the activation, maturation, and secretion of inflammatory factors such as IL-1*β* [7]. Four kinds of inflammatory corpuscles have been identified: nod-like receptor protein (NLRP) 1, NLRP3, IPAF, and AIM2. As the first discovered inflammasome, nucleotide-binding oligomerization domain-like NLRP1 is widely present in macrophages and plays a crucial role in oxidative stress [8]. It is composed of NLRP1 protein, ASC, and CASP1 [9]. The complex is essential for caspase activation, hence resulting in it being named an “inflammasome.” Autophagy is closely related to the activation of the inflammasome. Autophagy can inhibit the activation of the inflammasome. Autophagy can also play a regulatory role through nondependent inflammasome pathways [10, 11]. On the other hand, defective autophagy leads to the accumulation of damaged mitochondria and the production of ROS and the release of mitochondrial DNA into the cytoplasm, which can trigger the activation of inflammasomes [12, 13]. Compared with NLRP1, more research has been applied to NLRP3, which is mainly composed of NLRP3 protein, CASP1, and ASC. However, whether the activation of NLRP1 inflammasomes is related to autophagy remains unclear. Therefore, based on determining whether the activation of NLRP1 inflammatory corpuscles is related to oxidative stress, whether NLRP1 inflammatory corpuscles can be degraded through autophagy-selective receptors is worth investigating.

The study found that NLRP3 and IL-1*β* expression was significantly increased in the luteal phase endometrium in women with a history of abortion [14]. Moreover, studies have confirmed that the expression of the NLRP3 inflammasome is increased in the placenta of preeclampsia patients [15]. In vitro cell experiments demonstrated that normal trophoblast cells of early pregnancy could activate inflammasome and increase IL-1*β* expression under stimulation [16, 17]. This suggests that the abnormal inflammatory response caused by inflammasomes may be related to abortion or preeclampsia [18]. At present, the relationship between the NLRP1 inflammasome and autophagy is not clear. Abortion involves both maternal and fetal factors, and their primary and secondary roles in the pathogenesis of pregnancy diseases are still controversial. The NLRP1 inflammasome is a potential target for the treatment of pregnancy diseases [19].

Therefore, in the present study, we used HTR-8/SVneo cells to establish an in vitro model of oxidative damage of human placental trophoblasts. It was designed to explore whether the activation and excessive autophagy of NLRP1 inflammasomes occur in oxidative stress injury and determine whether there is a cascade relationship between these two processes.

## 2. Materials and Methods

### 2.1. Establishment of an Oxidative Stress Model of HTR-8/SVneo Cells [20]

HTR-8/SVneo cells (provided by Dr. Charles Graham, Queen's University of Canada) were cultured in DMEM/F12 medium (GIBCO, USA), 10% inactivated fetal bovine serum (GIBCO), and 1% penicillin-streptomycin (GIBCO) at 37°C and 5% CO_2_ until the cell logarithmic growth stage. Cells were plated onto a six-well culture plate at 1 × 10^5^ cells/well and cultured in an incubator at 37°C and 5% CO_2_. After allowing 24 hours for cell attachment, the HTR-8/SVneo cell oxidative stress model was established successfully after the final concentration of H_2_O_2_ (Sigma-Aldrich, USA) had reached 300 *μ*mol/L, and it was cultured for 3 hours.

The cell viability assay was performed after the culturing period had elapsed. The culture supernatant was removed and washed twice with PBS. Then, 110 *μ*L of CCK-8 working solution was added (prepared in advance at a volume ratio of CCK-8 solution to the culture medium of 1 : 10). This mixture was incubated at 37°C for 2 hours, and then, the absorbance (expressed as the optical density (OD)) value of each well at 450 nm was detected using an automatic enzyme scale. The cell survival rate was calculated using the following formula: survival rate (%) = (experiment group OD − blank pore OD)/(control group OD − blank pore OD) × 100%.

### 2.2. Detection of Inflammasomes in the Oxidative Stress Model by Western Blot

The NLRP1 activator muramyl dipeptide (MDP) (Selleck, USA) was then added at 100 *μ*mol for 24 hours to the oxidative stress model according to the preliminary experiment and the published studies [21–24]. The cell viability determination was the same as that in Section 2.1.

Total protein from HTR-8/SVneo cells was prepared with RIPA lysing buffer. The sample proteins (20 *μ*g) of the different groups were separated using 10% SDS-PAGE and transferred onto PVDF membranes. These membranes were incubated with a primary antibody, followed by incubation of the anti-rabbit IgG secondary antibody. Protein expression was detected with an enhanced chemiluminescence detection kit. GAPDH served as an internal control. The antibodies included those for IL-1*β* (Abcam, ab216995), pro-IL-1*β* (Abcam, ab216995), pro-CASP1 (Abcam, ab207802), CASP1 (Abcam, ab207802), Beclin-1 (Abcam, ab207612), LC3 (Abcam, ab51520), p62 (Abcam, ab211324), ATG5 (Abcam, ab108327), ATG7 (Abcam, ab52472), NLRP3 (Abcam, ab263899), NLRP1 (BioLegend, 679802), and GAPDH (Abcam, ab128915). An ECL chemiluminescent reagent and imaging system (Bio-Rad, USA) were used to display protein bands, with the collected images analyzed using Bio-Rad software.

### 2.3. Observation of Autophagy Protein in Oxidative Stress by Immunofluorescence

The HTR-8/SVneo cells used to establish the oxidative stress model were fixed by adding 4% paraformaldehyde, and PBS was dropped into the cell plate holes according to a certain proportion of the first antibody (ab51520, LC3, red, Abcam; ab207612, Beclin-1, green, Abcam) followed by incubation at 4°C overnight. The next day, the covering tissues of the species corresponding to the first and second antibodies were dripped onto a circular area and incubated at room temperature for 1 hour in darkness. DAPI dye solution was dripped into the circle and incubated in the dark at room temperature for 15 min. Finally, the cell side was sealed downward on the slide with an antifluorescence quenching sealing agent. The sections were observed and imaged under laser scanning confocal microscopy (1000x) (Nikon, Japan).

### 2.4. Detection of Autophagy Proteins in Cells after NLRP1 Gene Silencing

#### 2.4.1. Design and Synthesis of siRNA

HTR-8/SVneo cells were divided into three groups: blank, siRNA-NC, and siRNA-NLRP1 groups. The siRNA was processed as described below (Table 1). Two hours before transfection, DMEM/F12 medium without serum was replaced. siRNA (10 *μ*L) was diluted with 100 *μ*L of serum-free Opti-MEM (at a concentration of 20 *μ*mol). The mixture was gently mixed with the pipette gun head and kept at room temperature for 5 min. Before use, Lipofectamine™ 2000 was gently mixed, and then, 5 *μ*L of Lipofectamine™ 2000 was diluted in 100*μ*L of Opti-MEM and let to stand for 5 min at room temperature. Lipofectamine™ 2000 and plasmid diluent (total volume of 200 *μ*L) were then mixed gently and left to stand at room temperature for 20 min. The mixed solution (200 *μ*L) was added to the culture in each cell plate hole, with the cell culture plate shaken gently before and after to mix the mixed solution with the culture medium in the culture plate. The cells were then cultured in an incubator at 37°C and 5% CO_2_. The mixed solution was sucked out after 6 hours and transferred to a normal medium. The culture was continued for 24 hours, and then, the total RNA of the cells was extracted, with the transfection efficiency detected using real-time fluorescence quantitative PCR (qRT-PCR).

#### 2.4.2. qRT-PCR

The total RNA was extracted according to the manufacturer's instructions for Trizol (Ambion, USA). The following two-step process was used to amplify the program on an ABI PRISM 7500 (ABI, USA) PCR system: (1) reverse transcription cDNA: 25°C for 5 min, 50°C for 15 min, 85°C for 5 min, and 4°C for 10 min, and (2) qRT-PCR: 50°C for 2 min and 95°C for 10 min, followed by 40 cycles of 95°C for 30 sec and 60°C for 30 sec. The relative expression of the sample was calculated quantitatively using the formula 2^–ΔΔCt^. PCR primers and internal reference GAPDH were synthesized by TSINGKE™ (China), as listed in Table 2.

#### 2.4.3. Expression of Inflammasomes and Autophagy Proteins after Silencing of NLRP1

Total protein from HTR-8/SVneo cells silencing NLRP1 protein was prepared with RIPA lysing buffer. The next western blot steps were the same as those in Section 2.2.

#### 2.4.4. Observation of Autophagy Protein in Oxidative Stress by Immunofluorescence after Silencing of NLRP1

The immunofluorescence steps were the same as those in Section 2.3.

### 2.5. Statistical Analyses

All data are expressed as mean ± SD, obtained from more than three independent experiments, and analyzed by GraphPad Prism 9.0 (GraphPad Software, CA, USA). Statistically significant differences (^∗^*P* < 0.05, ^∗∗^*P* < 0.01) were examined using Student's *t*-test and one-way ANOVA.

## 3. Results

### 3.1. Establishment of an Oxidative Stress Model of HTR-8/SVneo Cells

**Figure 1:**
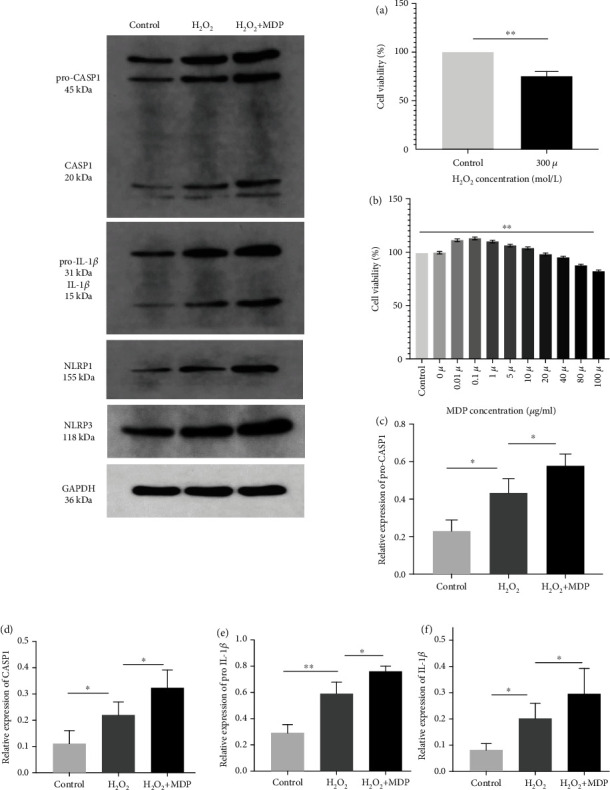
(a) The concentration of H_2_O_2_ on HTR-8/SVneo cell line activity using the CCK-8 method (^∗∗^*P* < 0.01). (b) The concentration of MDP on HTR-8/SVneo cell line activity using the CCK-8 method (^∗∗^*P* < 0.01). (c–f) Effects on intracellular inflammasome levels in the HTR-8/SVneo cell oxidative stress model: the expression levels of pro-CASP1 (c), CASP1 (d), pro-IL-1*β* (e), and IL-1*β* (f) were significantly higher in the model group than in the control group. After adding MDP, the expression levels of pro-CASP1, CASP1, pro-IL-1*β*, and IL-1*β* were significantly higher in the MDP group than in the model group (^∗^*P* < 0.05, ^∗∗^*P* < 0.01). Values are means ± SD (*n* = 3 per group).

As shown in Figure 1, the survival rate of HTR-8/SVneo cells was 76.78% after treatment with H_2_O_2_ at 300 *μ*mol/L beyond 3 hours compared with the control group (*P* < 0.01). These findings indicating the presence of both cell damage and cell viability met the experimental research conditions' requirements. So this was selected as the standard condition in subsequent experiments. Simultaneously, 24 hours after the addition of NLRP1-specific agonist MDP, the cell viability of 100 *μ*mol/L was 82.44% (*P* < 0.01), which best met the experimental requirements. Therefore, this concentration was selected as the experimental concentration for subsequent experiments.

### 3.2. Expression of Inflammasomes in the Oxidative Stress Model

**Figure 2:**
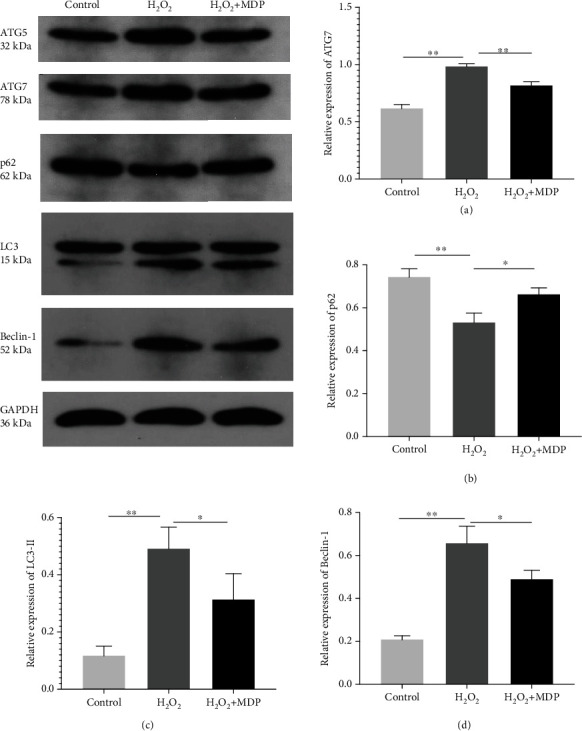
Autophagy-related protein expression in an HTR-8/SVneo cell oxidative stress model was detected by western blot: (a) ATG7, (b) p62, (c) LC3-II, and (d) Beclin-1 (^∗^*P* < 0.05, ^∗∗^*P* < 0.01). Values are means ± SD (*n* = 3 per group).

**Figure 3:**
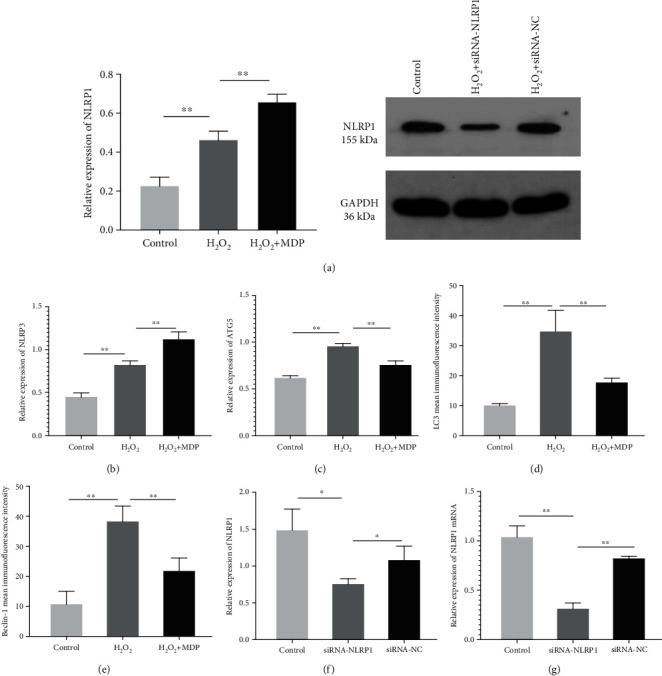
(a, b) Effects on intracellular inflammasome levels in the HTR-8/SVneo cell oxidative stress model: the expression levels of NLRP1 (a) and NLRP3 (b) (^∗^*P* < 0.05, ^∗∗^*P* < 0.01). (c) Autophagy-related protein expression levels of ATG5 (^∗^*P* < 0.05, ^∗∗^*P* < 0.01). (d, e) Average immunofluorescence intensity of autophagy-associated proteins, LC3 (d) and Beclin-1 (e) (^∗^*P* < 0.05, ^∗∗^*P* < 0.01). (f, g) The expression levels of the NLRP1 gene in HTR-8/SVneo cells transfected with siRNA-NLRP1 were detected by western blot (f) and real-time fluorescent quantitative PCR (g). The results showed that the expressions of NLRP1 and mRNA level were significantly downregulated after siRNA-NLRP1 (^∗^*P* < 0.05, ^∗∗^*P* < 0.01, vs. the control and the siRNA-NC groups). Values are means ± SD (*n* = 3 per group).

After successfully establishing a cellular oxidative stress model, we used it to investigate inflammasome activation and NLRP1 protein expression. Western blotting was used to detect inflammasomes' expression in the control group, model group, and MDP model. The results showed that the expression of NLRP1 and NLRP3 in the oxidative stress model of HTR-8/SVneo cells treated with H_2_O_2_ was significantly increased (*P* < 0.01). The NLRP1 activator can also significantly increase the expression of NLRP1 and NLRP3 protein (*P* < 0.01). NLRP1 plays an important role in the pathogenesis of inflammation. The expression levels of pro-CASP1, CASP1, pro-IL-1*β*, and IL-1*β* in the model and control groups were further detected, which revealed that they were significantly higher in the model group than in the control group (*P* < 0.01). Besides, after adding MDP, the expression levels of pro-CASP1, CASP1, pro-IL-1*β*, and IL-1*β* were significantly higher in the MDP group than in the model group (*P* < 0.01) (Figures 1–3).

### 3.3. Expression of Autophagy in Oxidative Stress

**Figure 4:**
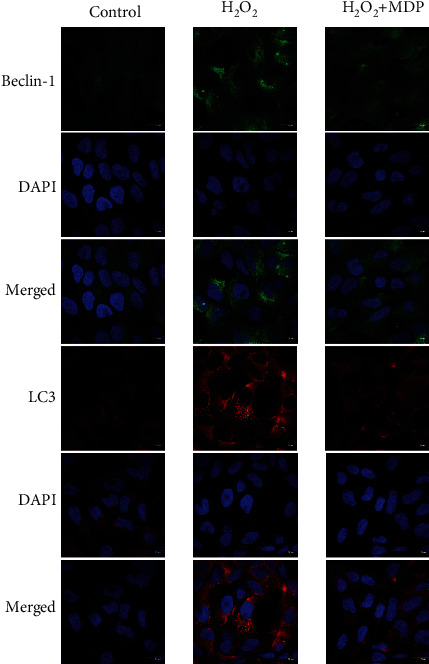
Autophagy-related protein expression in an HTR-8/SVneo cell oxidative stress model in immunofluorescence. Rabbit anti-LC3 monoclonal antibody (red) and DAPI staining were used to display nuclear (blue) immunolabeling. Scale: 20 *μ*m. Nuclear DAPI staining (blue) with rabbit anti-Beclin-1 monoclonal antibody (red) immunostaining microscopy. Scale: 20 *μ*m.

As shown in Figures 3 and 4, to confirm that LC3 and Beclin-1 are autophagic central proteins expressed in the H_2_O_2_-induced oxidative stress model of HTR-8/SVneo cells, we labeled LC3 and Beclin-1 in cells with red fluorescence and labeled their nuclei with blue fluorescence (using DAPI staining). The distribution of LC3 and Beclin-1 in cells was observed using laser scanning confocal microscopy. In the HTR-8/SVneo cell oxidative stress model, many fluorescence spots were evident in the cytoplasm, which indicated an elevation of the autophagy bodies, and the expression level of autophagy regulatory protein increased significantly. In contrast, there were only weak fluorescent spots in the control group. The fluorescence intensity can reflect the autophagy level. So, the above results show that oxidative stress and a high level of autophagy expression were present in the placenta trophoblasts (*P* < 0.01). After adding MDP, the expression level of autophagy regulatory protein decreased significantly (*P* < 0.01).

We used it to investigate the expression of autophagy-related protein expression. Western blot results (Figures 2 and 3) showed that the expression of LC3-II, Beclin-1, ATG5, and ATG7 in the oxidative stress model of HTR-8/SVneo cells treated with H_2_O_2_ was significantly increased as compared to that in the control group (*P* < 0.01) and the expression of p62 was significantly decreased as compared to that in the control group. After adding MDP, these proteins' expression levels were shown the opposite results in the MDP group than in the model group (*P* < 0.01).

### 3.4. siRNA Silence after HTR-8/SVneo Cell NLRP1 Gene Changes of Its Protein and mRNA Expression

Western blot and qRT-PCR results (Figure 3) show that NLRP1 protein and mRNA expression of siRNA-NLRP1 group cells is relatively lower, significantly lower than that of the siRNA-NC group and control group to 70% (*P* < 0.01).

### 3.5. Autophagy in an HTR-8/SVneo Oxidative Stress Model Is Associated with the NLRP1 Inflammasomes

**Figure 5:**
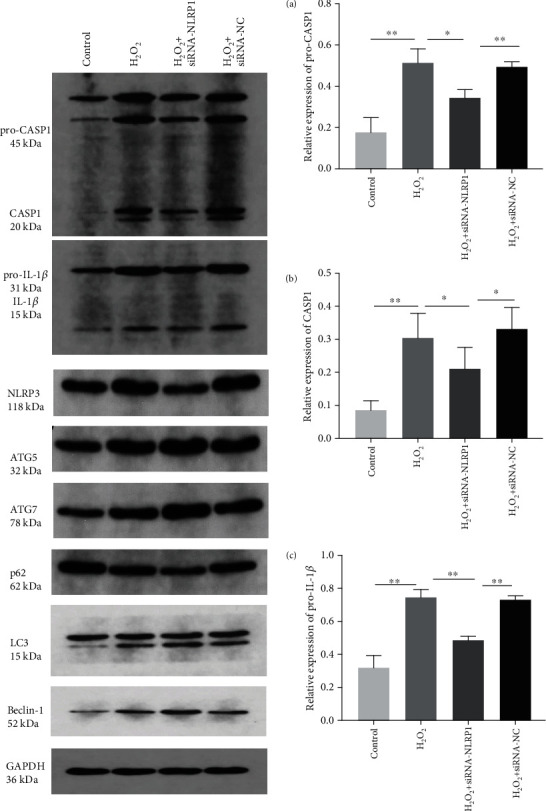
Effects of silencing the NLRP1 gene on autophagy-inflammasome levels in HTR-8/SVneo cells. (a–c) The expression levels of pro-CASP1 (a), CASP1 (b), and pro-IL-1*β* (c) (^∗^*P* < 0.05, ^∗∗^*P* < 0.01). Values are means ± SD (*n* = 3 per group).

**Figure 6:**
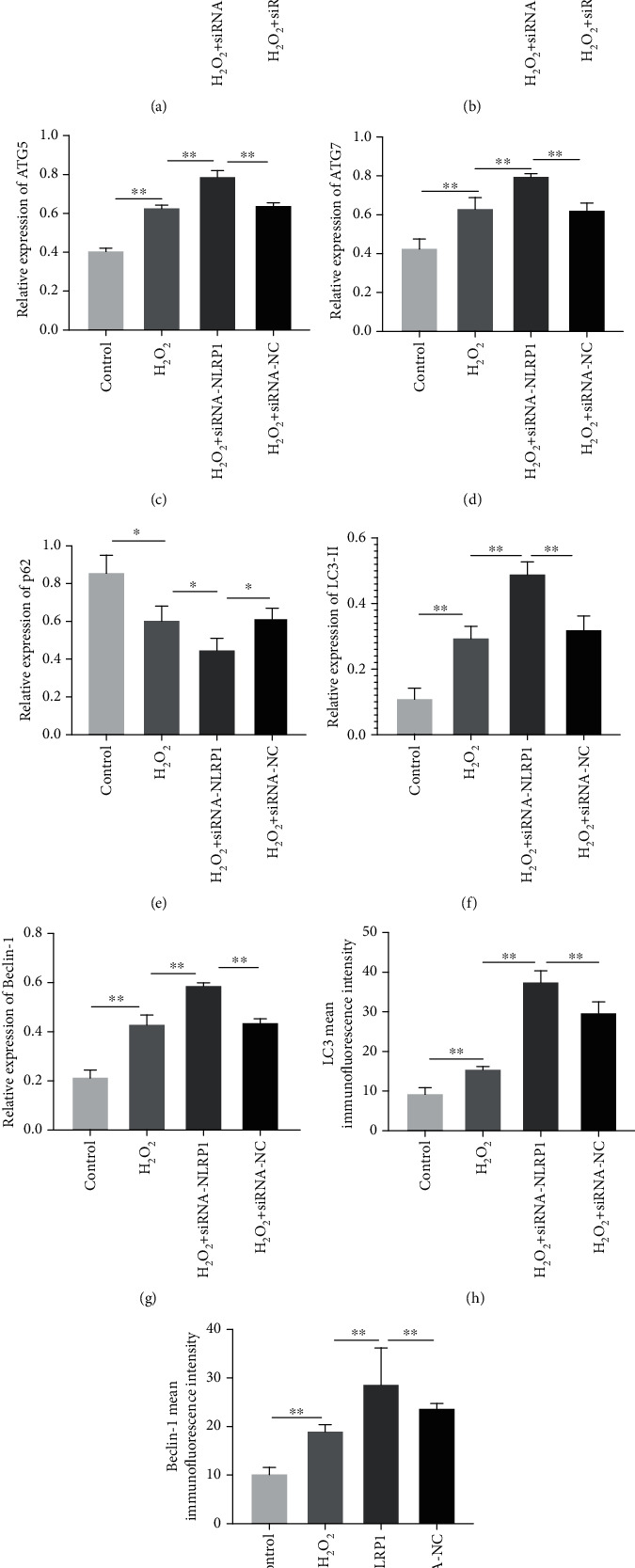
(a–g) The expression levels of IL-1*β* (a), NLRP3 (b), ATG5 (c), ATG7 (d), p62 (e), LC3-II (f), and Beclin-1 (g) of silencing the NLRP1 gene on autophagy-inflammasome levels in HTR-8/SVneo cells. (h, i) Average immunofluorescence intensity of autophagy-associated proteins, LC3 (h) and Beclin-1 (i) (^∗^*P* < 0.05, ^∗∗^*P* < 0.01). Values are means ± SD (*n* = 3 per group).

To further clarify the cascade relationship between the activation of NLRP1 inflammatory corpuscles and excessive autophagy of trophoblasts in an oxidative stress injury state, we transfected HTR-8/SVneo cells with siRNA-NLRP1 for 24 hours to establish the oxidative stress model of placental cells. As shown in Figures 5 and 6, western blot was used to observe the expression levels of LC3, Beclin-1, ATG5, ATG7, NLRP3, p62 pro-CASP1, CASP1, pro-IL-1*β*, and IL-1*β*. The results showed that H_2_O_2_ significantly increased the expression levels of LC3, Beclin-1, ATG5, ATG7, and NLRP3 in the cells (*P* < 0.01) and also those of pro-CASP1, CASP1, pro-IL-1*β*, IL-1*β*, and other inflammatory factors (*P* < 0.01) and decreased the expression levels of p62 (*P* < 0.01).

Besides, siRNA-NLRP1 could significantly inhibit the expression levels of NLRP3, p62, pro-CASP1, CASP1, pro-IL-1*β*, and IL-1*β* (*P* < 0.01), and siRNA-NLRP1 could significantly increase the expression levels of LC3, Beclin-1, ATG5, and ATG7 (*P* < 0.01). However, there was no significant difference in protein expression between the siRNA-NC and control groups. These results show that siRNA-NLRP1 can effectively reduce the expression level of NLRP1 and its family members, NLRP3, and that inhibition of NLRP1 activation under oxidative stress can instead increase the level of autophagy in a compensatory manner (Figures 5 and 6).

**Figure 7:**
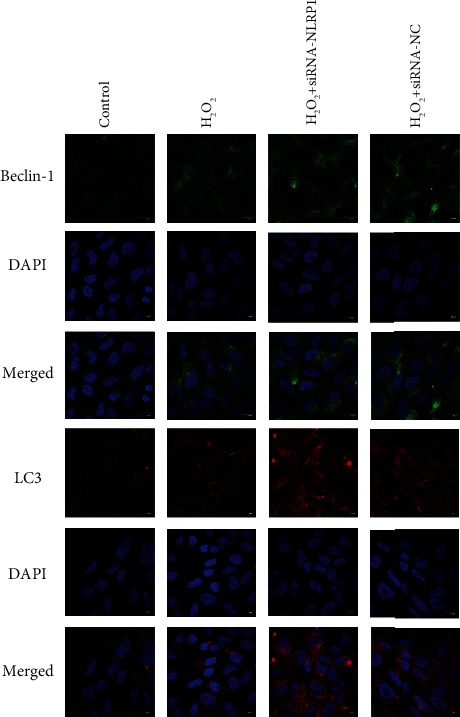
Autophagy-related protein expression in an HTR-8/SVneo cell oxidative stress model in immunofluorescence silencing the NLRP1 gene. Rabbit anti-LC3 monoclonal antibody (red) and DAPI staining were used to display nuclear (blue) immunolabeling. Scale: 20 *μ*m. Nuclear DAPI staining (blue) with rabbit anti-Beclin-1 monoclonal antibody (red) immunostaining microscopy. Scale: 20 *μ*m.

Besides, consistent with the western blot analysis, the expression of LC3 and Beclin-1 in the siRNA-NLRP1 HTR-8/SVneo cells was higher by immunofluorescence compared with that in the H_2_O_2_ model group (Figures 6 and 7).

## 4. Discussion

Pregnancy is a complex process whose success requires the maternal immune system not to reject the fetus as an allogeneic graft. Early-pregnancy trophoblasts perform unique physiological functions. The normal development of trophoblast proliferation and invasion is significant for blastocyst implantation, placenta formation, and the correct mother-fetus relationship. Studies have demonstrated that pregnancy diseases are related to the impairment or defect of trophoblast function. Also, abnormal changes in apoptosis and autophagy are essential factors promoting the impairment or deficiency of trophoblast function that results in defective placental formation or its shallow immersion. Inadequate placental perfusion, ischemia, and hypoxia may result in the body producing many ROS, further inducing the death of a large number of trophoblasts and eventually leading to pregnancy diseases and other pathological damage [25]. To clarify the biological relationship between H_2_O_2_-induced autophagy and activation of the NLRP1 inflammasome, our results clearly show that we established the oxidative stress model of HTR-8/SVneo cells induced by H_2_O_2_, suggesting that H_2_O_2_ is a reasonable inducer of oxidative stress. It has been found that ROS plays a vital role in the activation of inflammasomes. As a response to ROS, thioredoxin can bind directly to NLRPs. ROS regulates inflammasome activation mainly through autophagy [26]. Activation of the inflammasome and increase in synthesis of proinflammatory factors such as IL-1*β* can inhibit autophagy, and decrease in expression of the NLRP inflammasome can significantly improve autophagy ability of cells and thus inhibit oxidative stress-induced apoptosis and inflammation [27]; in the meantime, autophagy can also play a regulatory role through nondependent inflammasome pathways and inhibit inflammasome activation.

The NLRP protein complexes comprise members of the NLRP protein family, CASP1, and apoptosis-associated proteins containing a caspase-activating acceptance domain (e.g., ASC). NLR inflammasomes are the most well-studied inflammasomes and include NLRP1, NLRP3, and NLRC4 [28]. NLRP1 is one of the earliest members of the nod-like receptor (NLR) family [29]. NLRP1 and its effector CASP1 are critical upstream pathways of IL-1*β* maturation. External stimuli can result in the activated NLRP1 hydrolyzing the pro-CASP1 molecule in its constituent structure to produce mature CASP1, release mature CASP1 in large quantities, decompose pro-IL-1*β* (which is not active) into activated IL-1*β*, and participate in the inflammatory process [30]. NLRP3 is also an essential member of the NOD-like receptor family. After activation of the NLRP3 inflammasome, IL-1*β* is released to promote insulin resistance and induce *β*-cell functional impairment and apoptosis of the inflammasome. NLRP3 is the most thoroughly studied inflammasome [31]. In vitro cell experiments have demonstrated that normal trophoblast cells of early pregnancy can activate the NLRP3 inflammasome under stimulation and increase IL-1*β* expression [17]. This suggests that NLRP3 inflammasomes can also cause abnormal inflammatory responses related to abortion or preeclampsia. Therefore, for this study's rigor, we simultaneously monitored the expression of two inflammasomes in this study. The results showed that, as expected, the expression of NLRP3 was changed with the change of NLRP1.

Human NLRP1 inflammasomes can be activated explicitly by acyl dipeptides (e.g., MDP) in the bacterial cell wall [32]. In vitro studies have shown that MDP can activate NLRP1 inflammatory corpuscles [33], activate CASP1, enhance the transcription of IL-1*β* precursor, and ultimately accelerate the secretion of proinflammatory factors to participate in and regulate the inflammatory response [34, 35]. The level of CASP1 is influenced by MDP activating NLRP1 [21]. At the same time, it was found that NLRs had an essential effect on the expression of IL-1*β* [36] and that the expression of IL-1*β* decreased significantly when NLRP-deficient macrophages were stimulated by MDP [21]. Therefore, the level of CASP1 and the release of IL-1*β* are significant in promoting apoptosis and inflammation. We used H_2_O_2_ to establish an oxidative stress model of HTR-8/SVneo cells in vitro. Compared with the control group, the expression level of NLRP1 and NLRP3 inflammasomes increased significantly, which caused an increase in the CASP1 level, induced apoptosis, and induced the expression level of IL-1*β*—a significant proinflammatory factor—to increase significantly. The expression levels of pro-IL-1*β* and pro-CASP1 protein also increased. After the addition of MDP, which is an NLRP1-specific ligand, the level of these proteins increased further, indicating that H_2_O_2_ activated the inflammasomes NLRP1 and NLRP3 in placental trophoblasts and promoted the release of IL-1*β* via the CASP1 pathway; increases in inflammasomes are not unexpected as MDP is a known initiator of inflammation. MDP can activate NLRP1, which is involved primarily in inflammasome activity typically measured via IL-1*β* output [33]. Simultaneously, after we silenced the expression of NLRP1, oxidative stress was also weakened, which was specifically manifested as the decreased expression of IL-1*β* and CASP1, which was consistent with the previous results [37].

Autophagy is an important metabolic process of the body. In order to meet the needs of cell metabolism and maintain the homeostasis of the internal environment, the desquamated endoplasmic reticulum can wrap aging and damaged or abnormal biomacromolecules and organelles to form a double-membrane structure of autophagy bodies. The degradation of lysosomes will generate new energy substances for use in cell recycling. However, under pathological conditions, excessive autophagy can damage the proteins and organelles in cells and cause cell death [38]. Beclin-1 and LC3 play important roles in different stages of autophagy [39]. LC3-II is a marker protein of autophagy. Autophagy maturation involves LC3-I transforming into LC3-II [40], and so the expression level of LC3-II protein can reflect the autophagy level [41]. Beclin-1 is also a key protein for autophagy, and it can induce other autophagy genes to locate in the autophagy membrane and regulate the formation of autophagy bodies [42]. The expression level of Beclin-1 is positively correlated with the autophagy level, which is also an important basis for detecting autophagy [43]. At the molecular level, the ASC component of the NLRP1 inflammasome is ubiquitinated and recognized by the adaptor protein p62. p62 (also known as sequestosome 1/SQSTM1) is a well-known substrate of selective autophagy. p62 can directly interact with LC3 on the separation membrane through the LC3 interaction region. p62 is then recruited into the autophagosome and degraded. Lack of autophagy leads to the accumulation of p62, leading to the formation of large aggregates, including p62 and ubiquitin. Besides, p62 acts as an adaptor by interacting with the autophagosome localizing LC3 and ubiquitin chains on damaged organelles [44]. The formation of autophagy vacuoles is mediated by up to 30 autophagy-related proteins encoded by the ATG gene, originally identified in yeast [45]. More than 40 genes encoding autophagy-associated proteins (ATG proteins) have been identified, of which more than 15 are the core genes of hunger-induced autophagy. Autophagy-related proteins are mostly conserved in other organisms, including mammals, suggesting that autophagy is an evolutionarily conserved process. Mammals have some autophagy genes such as ATG5, Beclin-1 (ATG6), ATG7, and LC3 (ATG8) [46, 47]. LC3 cleans C-terminal glycine residue from protease ATG4 and binds to phosphatidylethanolamine through ATG7, ATG3 and ATG5-ATG12-ATG16L1 complexes [48]. It was suggested that LC3-phosphatidylethanolamine mediated the tethering and semiperfusion of the cell membrane to form autophagosomes. ATG3, ATG5, ATG7, or ATG16L1 deficiency in mice leads to malnutrition and energy expenditure, leading to death shortly after birth [49].

Autophagy and apoptosis are induced by many factors and have a complicated relationship. One of the more important factors is an imbalance of ROS generation and clearance [50]. ROS is a direct primer for oxidative stress [6, 51]. Many studies have demonstrated that ROS produced in oxidative stress can induce autophagy [6, 52]. H_2_O_2_ can prevent the decrease in LC3 and promote the formation of autophagy bodies, thus promoting autophagy activation. Autophagy can protect cell function and promote cell death. In most cases, trophoblast autophagy is inhibited at a basic level. Hypoxia and oxidative stress can significantly increase ROS content [53], the expression of autophagy-related protein LC3 in placental trophoblasts, and the autophagy level [54]. Oxidative stress not only enhances autophagy but also induces apoptosis and the inflammatory response [55]. The molecular mechanism regulating autophagy and inflammatory corpuscles in the oxidative damage of placental trophoblasts is unclear. It is necessary to determine whether autophagy is involved in the pathological process of placental dysfunction in pregnancy-related diseases, whether there is excessive autophagy in trophoblasts due to extensive oxidative stress injury, and what the underlying mechanisms are. The present study found that oxidative stress increased the expression of NLRP1 and also the autophagy level. The present study found that oxidative stress increased the expression of NLRP1, NLRP3, etc. and even the autophagy level. It is suggested that excessive oxidative stress may lead to the covalent regulation of protein structure and functional changes in trophoblasts, leading to increases in apoptosis and autophagy and finally changes to placental function [56]. There is increasing evidence that Beclin-1-mediated autophagy is related to placental dysplasia in HTR-8/SVneo cells [57]. However, inadequate or excessive autophagy of placental trophoblasts can cause trophoblastic dysfunction [58]. All of these observations support the presence of a close relationship between oxidative stress and autophagy. The application of H_2_O_2_ in establishing the model also indicates a common oxidative stress inducer, which is also essential in autophagy [6]. Cells treated with H_2_O_2_ can rapidly produce considerable amounts of ROS and then induce apoptosis.

Autophagy and inflammasome are functionally related. Both control homeostasis processes such as metabolism, energy production, organelle maintenance, and strict control of inflammation and pathogen clearance. Therefore, it is crucial to understand how autophagy and inflammasome mechanisms intersect and regulate. Activation of the autophagy and inflammasome complex is the physiological pathway that controls tissue homeostasis and immunity [59, 60]. Autophagy and inflammasome cross and regulate each other to maintain tissue homeostasis [61]. In addition, the occurrence and development of the disease are very complicated processes. Autophagy is closely related to the inflammatory process under many pathological conditions: autophagic proteins can activate or inhibit the inflammatory response [62], and inflammatory signals can induce or inhibit autophagy [38]. However, the mutual regulation mechanisms of autophagy and inflammasome are not fully understood [13, 38, 50].

It has been reported that there is an antagonistic mechanism between autophagy and inflammasome, suggesting that the inflammasome has an inhibitory effect on autophagy; inhibition of autophagy leads to abnormal activation of inflammasomes, leading to the development of inflammatory diseases [63]. This study showed that after the addition of the NLRP1 agonist MDP to the model of cellular oxidative stress, both the oxidative stress level and the inflammation level were overactivated. The expression of NLRP3 was also increased, but instead, autophagy is inhibited, the levels of Beclin-1, ATG5, and ATG7 and the ratio of LC3-II to LC3-I were significantly decreased, and the increase in p62 level indicated that autophagy was significantly inhibited in vivo, illustrating that the excessive activation of inflammatory corpuscle can lead to accumulation of damaged mitochondria and ROS production and release of mitochondrial DNA in the cytoplasm, leading to cell autophagy defects. *Pseudomonas aeruginosa* induced the activation of the NLRC4 inflammasome in macrophages. NLRC4 activates CASP1 and then directly cleaves TRIF (TIR domain-containing adaptor inducing interferon-*β*) to reduce TRIF-mediated autophagy [64]. Similarly, AIM2 and NLRP3 also negatively regulate autophagy through CASP1 release and inactivation of Parkin (an essential mitochondrial phagocytic protein), leading to expanded mitochondrial damage and focal erosion [65]. In addition, NLRP4 has also been reported to regulate autophagy by interacting with Beclin-1 negatively.

Autophagy can also negatively regulate the secretion of inflammasomes [10]. When inflammasomes are in an inferior position, autophagy can play a regulatory role, and autophagosomes can isolate pro-IL-1*β* and degrade it, thus reducing the activation substrate of caspase-1 inhibiting inflammasomes [11]. Through siRNA-NLRP1, we reduced the expression of NLRP1, NLRP3, IL-1*β*, and CASP1 protein in HTR-8/SVneo cells induced by H_2_O_2_. Simultaneously, the expression ratio of LC3-II relative to LC3-I and the expression of Beclin-1 (a positive regulatory factor of autophagy), ATG5, and ATG7 both increased, and p62 was decreased, which can significantly enhance the autophagy level of cells. Autophagy negatively regulates the activation of inflammasomes, thereby inhibiting the body's inflammatory response, which may have a highly harmful effect on cells and tissues [66]. The interaction between autophagy and inflammasome is mutually regulated [13]. Autophagy of inflammasomes stimulates the formation of autophagosomes and the destruction of inflammasomes, limiting the production of IL-1*β* [60]. Resveratrol, a naturally occurring plant polyphenol compound, has been shown to inhibit the activation of the NLRP3 inflammasome in macrophages by inducing autophagy in a mouse model of progressive IgA nephropathy [67]. Berberine can also inhibit the activation of the NLRP3 inflammasome by upregulating the level of macrophage autophagy. Furthermore, decreasing Beclin-1 level or adding autophagy inhibitors can reverse the inhibition of the NLRP3 inflammasome by berberine [68]. ATG5 acetylation inhibits the overactivation of autophagy and the NLRP3 inflammasome in ATG5 acetylated cells. In contrast, SIRT3 activates autophagy, inhibits NLRP3 inflammasomes, and blocks endogenous ATG5 acetylation by forming complexes with ATG5 in the cell [69]. Therefore, it can be inferred that autophagy is negatively correlated with the inflammasome.

In conclusion, there are now a large number of published studies showing that inflammasomes and autophagy regulate each other. In most cases, this bidirectional regulation pathway seems to provide the necessary checks and balances between host defense against the inflammatory response and prevention of excessive inflammation that can lead to organ and tissue damage and inflammatory diseases. The results of this study have shown that H_2_O_2_ can induce oxidative stress in placental trophoblasts. That excessive amounts of ROS participate in the level of NLRP1 inflammasomes, promoting the secretion of IL-1*β* and CASP1 and leading to an inflammatory response of the body, thus enhancing trophoblast autophagy and inducing apoptosis; in turn, as the inflammasome weakens, autophagy returns to its prime position to ensure cell survival when the inflammasome is overactivated; autophagy is damaged. There is currently considerable interest in the relationship between autophagy and inflammatory corpuscles. An in-depth exploration of this relationship will help understand the physiological and pathological processes related to autophagy and inflammation. As we learn more about how the autophagy and inflammasome pathways are regulated separately, we seem to be discovering multiple layers of regulation of the inflammatory response. It is possible that the interaction of these essential pathways could control the fate of cells, helping them survive inflammatory damage or pushing them toward some form of cell death. Most of the results seem to depend on the cell types involved and the specific conditions that induce inflammation, inflammasome activation, and autophagy. This study has demonstrated a new way to investigate the pathogenesis of trophoblast autophagy, apoptosis, and pregnancy-related diseases under oxidative stress that we may in the future be able to manipulate to relieve and help millions of patients with pathologic pregnancies.

## Figures and Tables

**Table 1:** siRNA sequences in different experimental groups.

siRNA	Gene sequence
siRNA-NLRP1	5′-CCAAAUGGCCCACUUUAAATT-3′ 5′-UUUAAAGUGGGCCAUUUGGTT-3′
siRNA-NC	5′-UUCUCCGAACGUGUCACGUTT-3′ 5′-ACGUGACACGUUCGGAGAATT-3′

**Table 2:** Primer sequences.

Name	Primer	Sequence	Size
Homo GAPDH	Forward	5′-TCAAGAAGGTGGTGAAGCAGG-3′	115 bp
	Reverse	5′-TCAAAGGTGGAGGAGTGGGT-3′	
Homo NLRP1	Forward	5′-GATCAACCCACAGCACAGC-3′	210 bp
	Reverse	5′-TCCAGAACTATGTGATGCAGC-3′	

## Data Availability

All data generated or analyzed during this study are available from the corresponding author on reasonable request.

## Abbreviations

ATG5: Autophagy-related 5 (protein-containing ubiquitin folds)
ATG7: Autophagy-related 7 (ubiquitin-activating enzyme homologue)
Beclin-1: Mammalian homologue of yeast Atg6, activating macroautophagy
CASP1: Cysteine aspartic acid-specific protease 1
IL-1*β*: Interleukin 1, beta
ROS: Reactive oxygen species
DAPI: 40,6-Diamidino-2-phenylindole
H_2_O_2_: Hydrogen peroxide
LC3: Microtubule-associated protein light chain 3
MDP: Muramyl dipeptide
NLRP1: NLR family pyrin domain containing 1
NLRP3: NLR family pyrin domain containing 3
PBS: Phosphate-buffered saline
p62: Autophagy receptor p62
siRNA: Small interfering RNA
TRIF: TIR domain-containing adaptor inducing interferon-*β*.

## Additional Points

*Capsule.* The activation and overautophagy of NLRP1 inflammasomes in human placental trophoblastic oxidative stress and autophagy production are related to the activation of NLRP1 inflammasomes.
